# Utility of prognostic nutritional index and systemic immune-inflammation index in oral cancer treatment

**DOI:** 10.1186/s12885-022-09439-x

**Published:** 2022-04-07

**Authors:** Kosei Kubota, Ryohei Ito, Norihiko Narita, Yusuke Tanaka, Ken Furudate, Natsumi Akiyama, Chuang Hao Chih, Shotaro Komatsu, Wataru Kobayashi

**Affiliations:** 1 Department of Dentistry and Oral Surgery, Hirosaki University Graduate School of Medicine, 5 Zaifu-cho, Hirosaki, Aomori, 036-8562 Japan; 2 Department of Leukemia, The University of Texas MD Anderson Cancer Center, Houston, TX USA

**Keywords:** Prognostic nutritional index, Systemic immune-inflammation index, Inflammation-based prognostic score, Neutrophil-to-lymphocyte ratio, Lymphocyte-to-monocyte ratio, Platelet-to-lymphocyte ratio, Predictive factor for prognosis, Oral squamous cell carcinoma

## Abstract

**Purpose:**

This study aimed to evaluate the utility of inflammation-based prognostic scores (IBPS) and systemic immune-inflammation index (SII) in the treatment of oral cancer patients.

**Methods:**

For the 183 patients enrolled in this study, IBPS and SII were calculated from peripheral blood samples obtained before and after treatment and at the time of relapse. We examined overall survival (OS) and disease-free survival (DFS) using previously reported cut-off values for IBPS. Cut-off values of neutrophil-to-lymphocyte ratio (NLR), lymphocyte-to-monocyte ratio (LMR), platelet-to-lymphocyte ratio (PLR), and prognostic nutritional index (PNI) were analyzed as NLR 1.79, PLR 114.97, LMR 5, and PNI 52.44. The cut-off value for SII was set at 569. OS and DFS were analyzed by Kaplan–Meier methods using the cutoff of each IBPS and SII. Univariate analysis and multivariate analysis using Cox proportional hazards were performed for OS and DFS.

**Results:**

Kaplan–Meier methods showed the high-PNI group showed good prognosis including OS and DFS, while the high-SII group displayed poor DFS. Univariate analysis showed that pre-treatment high PNI and low SII were significantly associated with better prognosis. Multivariate analysis identified pre-treatment PNI as independently associated with OS. For DFS, univariate analysis using Cox proportional hazards modeling showed that pre-treatment high NLR and high SII were significantly associated with worse prognosis, while high PNI was significantly associated with better prognosis. Multivariate analysis identified pre-treatment PNI and SII as independently associated with DFS. Parameters of PNI and SII components were compared between pre-treatment, post-treatment and at relapse in the high- and low-PNI groups. PNI was predominantly decreased in both high- and low-PNI groups at post-treatment and at relapse compared to pre-treatment. This trend was also observed for albumin.

**Conclusions:**

Higher pre-treatment PNI was associated with better OS, while lower pre-treatment PNI and higher treatment SII were associated with poorer DFS in oral cancer patients. Our data indicated that PNI and SII might offer useful biomarkers for gauging prognosis and the efficacy of conventional therapies.

## Introduction

Treatments for oral squamous cell carcinoma include surgery, radiotherapy, chemotherapy, molecularly targeted drugs, and immune checkpoint inhibitors. Advances in treatment technologies have improved the effectiveness of treatment in the last decade, but oral and maxillofacial surgeons still encounter difficulty estimating prognosis. Biomarkers that can better predict prognosis and therapeutic response thus need to be developed to achieve more appropriate treatment. Inflammatory status has been implicated in the progression of cancer, and a number of inflammatory biomarkers that reflect the inflammatory status of cancer patients have been reported as prognostic factors in various carcinomas [1, 2]. Peripheral blood sampling is a simple and useful modality for measuring systemic inflammation in clinical situations. Using peripheral blood samples, lymphocyte-to-monocyte ratio (LMR), neutrophil-to-lymphocyte ratio (NLR), platelet-to-lymphocyte ratio (PLR) and prognostic nutritional index (PNI) have all been reported as inflammation-based prognostic scores (IBPS) for various cancers [3–7]. Recently, the systemic immune-inflammation index (SII), based on a combination of peripheral lymphocyte, neutrophil, and platelet counts, was hypothesized to better reflect the balance between host inflammation and immune status. The prognostic value of the SII in hepatocellular carcinoma, gastric cancer, colorectal cancer and small cell lung cancer has been confirmed, but few studies have focused on the importance of SII in oral cancer [8].

The purpose of this paper was to examine the prognostic values of different hematological IBPS such as NLR, PLR, LMR, PNI and SII and identify correlations with overall survival (OS) and disease-free survival (DFS) among patients with oral cancer treated by surgery, combination surgery and radiotherapy, and superselective intra-arterial chemoradiotherapy (SSIACRT).

## Patients and methods

### Patient characteristics

**Fig. 1:**
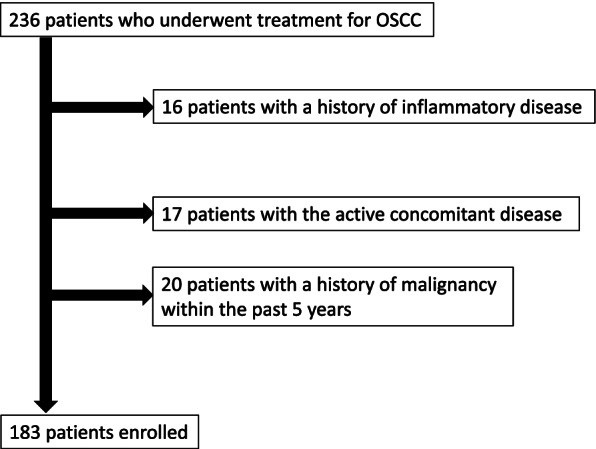
Flow diagram of cases enrolled in this study

We conducted a retrospective analysis of 183 patients who had received surgery, combination surgery and radiation therapy, or SSIACRT between 2005 and 2017 in the Department of Dentistry and Oral Surgery at Hirosaki University Hospital, Japan. Criteria for eligibility were measurable, histologically confirmed squamous cell carcinoma of the tongue, mandibular gingiva, maxillary gingiva, floor of the mouth, buccal mucosa, hard palate, or lip without any evidence of distant metastasis, and with World Health Organization performance status 0 or 1. No patients had received prior chemotherapy, radiotherapy (RT) or surgery. Fifty-three patients with a history of inflammatory disease, active concomitant infection, or history of malignancy within the past 5 years were excluded (Fig. 1).

All patients included were assessed by computed tomography (CT), magnetic resonance imaging, and positron emission tomography/CT. Clinical stage was determined by clinical examination and these radiographic examinations. All patients were staged according to the 7^th^ edition of the International Union against Cancer criteria [9].

Treatments of oral cancer patients were radical surgery with/without reconstruction (118 cases), radical surgery followed by adjuvant chemoradiotherapy/radiotherapy (31 cases), and SSIACRT (34 cases).

Contents of RT were as follows. The primary tumor and all nodal areas were irradiated with 50 Gy in 25 fractions, at 5 fractions a week, over a period of 5 weeks, immediately followed by a boost of 16 Gy in 8 fractions to all involved areas, including the primary tumor (total dose, 66 Gy).

In adjuvant chemoradiotherapy, identical RT plus concurrent intravenous cisplatin at 100 mg/m^2^ was administered on days 1, 22, and 43 [10].

SSIACRT patients received 3 infusions of concurrent intra-arterial docetaxel (40 mg/mm^2^) and nedaplatin (80 mg/mm^2^) every 4 weeks, as reported by Kobayashi et al. [11].

The ethics committee at Hirosaki University Graduate School of Medicine approved this study (approval no. 2018–1067) and waived the need to obtain informed consent, given the non-interventional retrospective design. All data were analyzed anonymously.

### Follow-up

Patients underwent hematological testing and assessment of clinical symptoms every month during the first year after treatment, and at intervals of 2 months during the second year, and intervals of 3–6 months thereafter until at least 5 years after treatment.

## Methods

Blood samples were collected at the time of initial diagnosis (pre-treatment), within 1 week of discharge (post-treatment), and within 1 week of confirmed relapse (relapse).

NLR was defined as the ratio of the number of neutrophils to the number of lymphocytes in blood. PLR was the ratio of the number of platelets to the number of lymphocytes, and LMR was the ratio of the number of lymphocytes to the number of monocytes. PNI was calculated using the following formula: 10 × serum albumin (g/dL) + 0.005 × total lymphocyte count in peripheral blood (/mm^3^) [3–5, 12]. SII was defined as platelet count × neutrophil count/lymphocyte count [8].

### Statistical analysis

Cut-off values were set as 1.79 for NLR, 5 for LMR, 114.97 for PLR, and 52.44 for PNI, referring to Watabe et al. [13]. The cut-off value for SII was set at 569, referring to Lu et al. [8]. Kaplan–Meier methods were used to estimate the probabilities of OS and DFS as a function of time, and statistical differences in OS and DFS of patient subgroups were compared using log-rank testing. To clarify prognostic factors for survival, Cox regression modeling was applied to identify the best predictors using uni- and multivariate analyses. Relative risks and 95% confidence intervals (CIs) were estimated for predictors using the regression models.

Multivariate analysis was performed based on clinical relevance and previous studies [13, 14], considering potential confounders such as age, sex, clinical tumor size, clinical lymph node metastasis, and treatment procedure.

NLR, LMR, PLR, PNI, and SII were compared among pre-treatment data, post-treatment data, and data at the time of relapse in the relapse group. Data for each assessment parameter were subjected to the Kruskal–Wallis test.

NLR, LMR, PLR, PNI, and SII were compared between pre- and post-treatment data in the death group. Data for each assessment parameter were subjected to Mann–Whitney U test.

Differences were considered significant for values of *p* < 0.05. All statistical analyses were performed using EZR [15], a modified version of R Commander designed to add statistical functions frequently used in biostatistics.

## Results

### Correlation and clinicopathological characteristics (Table 1)

**Table 1:** Correlation and clinicopathological characteristics

	Pre-treatment PNI < 52.4	Pre-treatment PNI ≥ 52.4	*p*
Age			
< 66	52	30	
≧66	89	12	0.0001*
Sex			
Male	77	26	
Female	64	16	0.48
cT			
cT1 + cT2	97	25	
cT3 + cT4	44	17	0.01*
cN			
cN0	104	26	
cN1 + cN2 + cN3	37	16	0.1
Treatment			
Surgery only	85	33	
Surgery + RT/CRT	26	5	
SSIACRT	30	4	0.1
Location			
Tongue	51	19	
Mandibular gingiva	35	8	
Maxillary gingiva	24	3	
FOM	18	5	
Buccal cheek mucosa	10	4	
Hard palate	3	2	
Lip		1	
Relapse			
Loco-regional relapse	33	3	
Distant relapse	11	2	
None	97	37	0.0163*
Survival status			
Alive	101	39	
Dead	40	3	0.0034*

*PNI* Prognostic nutritional index, *RT* Radiation therapy, *CRT* Chemoradiotherapy, *SSIACRT* Superselective intra-arterial chemoradiotherapy, *FOM* Floor of mouth

Median age was 66 years (range, 26–93 years). Participants comprised 103 men and 80 women. Tumor location was the tongue in 70 cases, mandibular gingiva in 43 cases, maxillary gingiva in 27 cases, floor of the mouth in 23 cases, buccal mucosa in 14 cases, hard palate in 5 cases, and lip in 1 case. Tumor stage was T1 in 41 patients, T2 in 82 patients, T3 in 21 patients, and T4 in 39 patients. Nodal stage was N0 in 130 patients, N1 in 16, N2 in 36, and N3 in 1. Classification of PNI by cut-off value was related to age (median, 66 years) and tumor size, neck metastasis, treatment, location, presence of recurrence, and survival. The frequency of low-PNI patients was significantly higher among those older than the median age, with advanced T stage, with relapse and among patients who died.

### Examination of OS and DFS using various IBPS and SII cutoff values

**Fig. 2:**
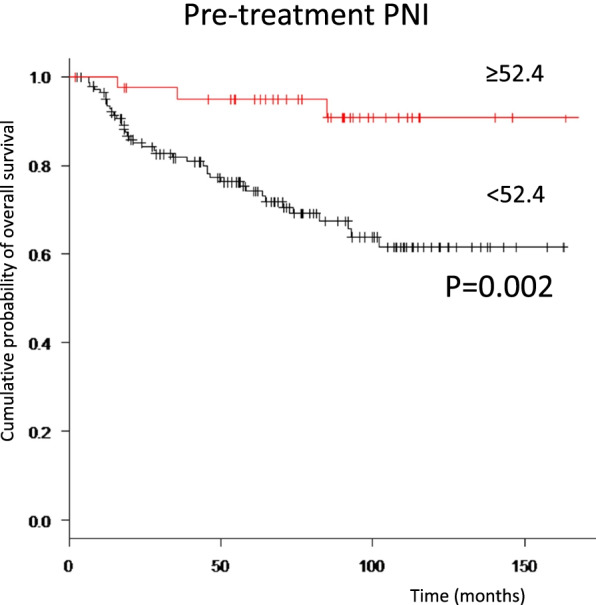
Relationships between pre-treatment PNI and overall survival in patients with oral squamous cell carcinoma. For Kaplan–Meier survival analyses, patients are divided into two groups based on PNI status (high-PNI and low-PNI groups). A significant difference is evident between groups (*p* = 0.002)

**Fig. 3:**
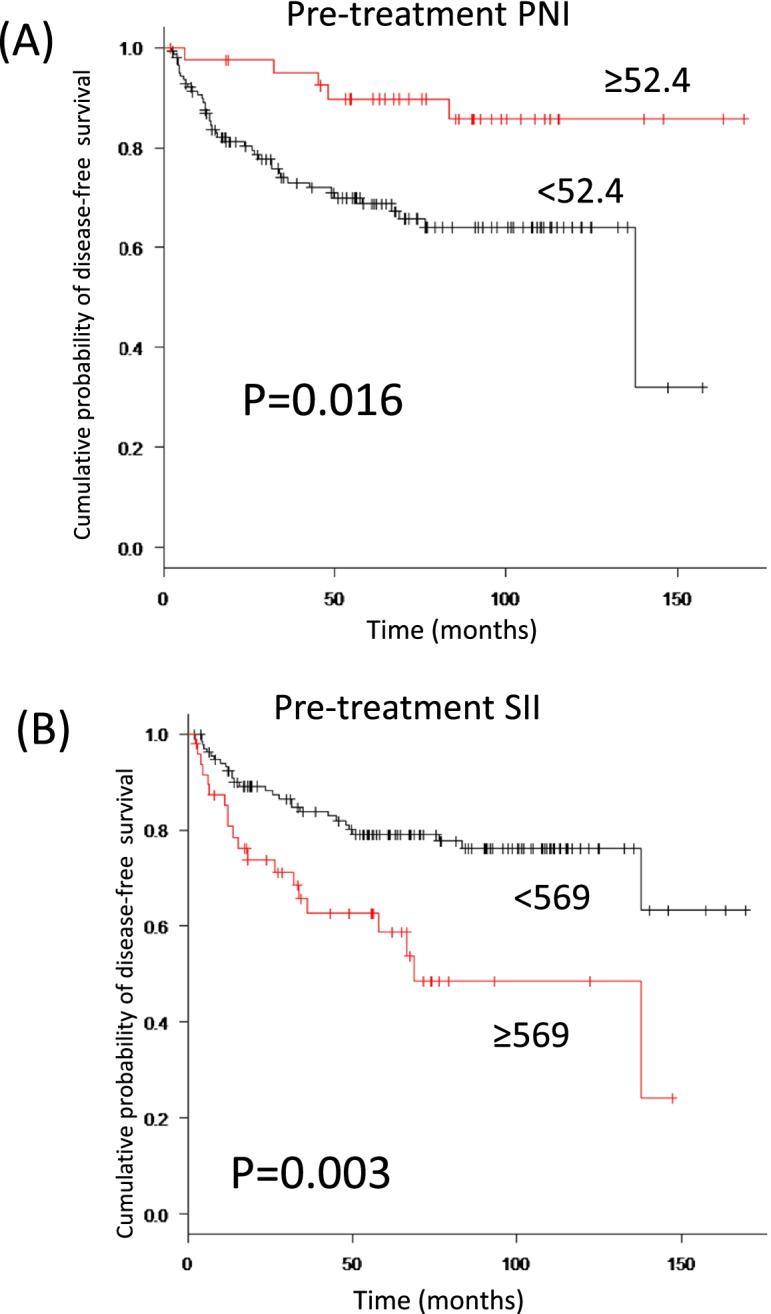
Relationships between pre-treatment PNI and disease-free survival in patients with oral squamous cell carcinoma. For Kaplan–Meier survival analyses, patients are divided into two groups based on PNI status (high-PNI and low-PNI groups). A significant difference is evident between groups (*p* = 0.016). For pre-treatment SII, disease-free survival is significantly worse in the high-SII group than in the low-SII group (*p* = 0.003)

The high-PNI group showed better prognosis, including OS and DFS (Figs. 2, 3A), while the high-SII group showed worse DFS (Fig. 3B).

### Uni- and multivariate analyses for OS (Table 2)

**Table 2:** Univariate and Multivariate analysis (Cox regression) for overall survival

Factor	Hazard.ratio	*p*.value
Univariate analysis (Cox regression) for overall survival		
Age(median.66)	5.32 (2.38–11.92)	0.0001*
Sex	0.79 (0.42–1.49)	0.48
cT1.2/3.4	0.56 (0.25–1.22)	0.14
cN0/1.2.3	1.88 (0.90–3.92)	0.094
Surgery(+ RT)/SSIACRT	1.39 (0.48–4.01)	0.55
Pre-treatment LMR < 5 ≥ 5	0.99 (0.48–2.06)	0.98
Pre-treatment NLR < 1.79 ≥ 1.79	0.50 (0.21–1.22)	0.13
Pre-treatment PLR < 114.97 ≥ 114.97	0.86 (0.40–1.88)	0.71
Pre-treatment PNI < 52.4 ≥ 52.4	0.29 (0.09–0.97)	0.044*
Pre-treatment SII < 569 ≥ 569	3.28 (1.29–8.30)	0.012*
Multivariate analysis (Cox regression) for overall survival		
Age(median.66)	3.8 (1.79–8.02)	0.0004*
Pre-treatment PNI < 52.4 ≥ 52.4	0.26 (0.08–0.86)	0.026*

*RT* Radiation therapy, *SSIACRT* Superselective intra-arterial chemoradiotherapy, *LMR* Lymphocyte-to-monocyte ratio, *NLR* Neutrophil-to-lymphocyte ratio, *PLR* Platelet-to-lymphocyte ratio, *PNI* Prognostic nutritional index, *SII* Systemic immune-inflammation index

In univariate analyses of pre-treatment parameters, none of sex, T stage, neck metastasis, treatment procedure (operation with or without RT/SSIACRT), LMR, NLR, and PLR were associated with OS. Age, PNI and SII were identified as predictors of OS (age: hazard ratio (HR) 5.32, 95%CI 2.38–11.92, *p* = 0.0001; PNI: HR 0.29, 95%CI 0.09–0.97, *p* = 0.044; SII: HR 3.28, 95%CI 1.29–8.30, *p* = 0.012). Multivariate analysis identified age and PNI as independent prognostic factors for OS (age: HR 3.8, 95%CI 1.79–8.02, *p* = 0.0004; PNI: HR 0.26, 95%CI 0.08–0.86, *p* = 0.026).

### Uni- and multivariate analyses for DFS (Table 3)

**Table 3:** Univariate and Multivariate analysis (Cox regression) for disease survival

Factor	Hazard.ratio	*p*.value
Univariate analysis (Cox regression) for disease free survival		
Age (median.66)	1.32 (0.7–2.47)	0.39
Sex	1.01 (0.55–1.86)	0.97
cT1.2./3.4	0.77 (0.37–1.58)	0.47
cN0/1.2.3	1.17 (0.57–2.41)	0.67
Surgery(+ RT)/SSIACRT	0.70 (0.24–2.00)	0.5
Pre-treatment LMR < 5 ≥ 5	1.37 (0.72–2.61)	0.33
Pre-treatment NLR < 1.79 ≥ 1.79	0.37 (0.14–0.96)	0.04*
Pre-treatment PLR < 114.97 ≥ 114.97	0.83 (0.39–1.76)	0.63
Pre-treatment PNI < 52.4 ≥ 52.4	1.57 (0.83–2.94)	0.014*
Pre-treatment SII < 569 ≥ 569	5.06 (1.85–13.88)	0.0016*
Multivariate analysis (Cox regression) for disease free survival		
Pre-treatment PNI < 52.4 ≥ 52.4	0.30(0.12–0.81)	0.016*
Pre-treatment SII < 569 ≥ 569	4.1(1.63–10.32)	0.003*

*RT* Radiation therapy, *SSIACRT* Superselective intra-arterial chemoradiotherapy, *LMR* Lymphocyte-to-monocyte ratio, *NLR* Neutrophil-to-lymphocyte ratio, *PLR* Platelet-to-lymphocyte ratio, *PNI* Prognostic nutritional index, *SII* Systemic immune-inflammation index

In univariate analyses of pre-treatment parameter, none of sex, T stage, neck metastasis, treatment procedure (operation with or without RT/SSIACRT), LMR, and PLR were associated with OS. NLR, PNI and SII were identified as predictors of DFS (NLR: HR 0.37, 95%CI 0.14–0.96, *p* = 0.04; PNI: HR 1.57, 95%CI 0.83–2.94, *p* = 0.014; SII: HR 5.06, 95%CI 1.85–13.88, *p* = 0.0016). Likewise, multivariate analysis identified age, PNI, and SII as independent prognostic factors for OS (PNI: HR 0.30, 95%CI 0.12–0.81, *p* = 0.016; SII: HR 4.1, 95%CI 1.63–10.32, *p* = 0.003).

### Comparison of pre-treatment, post-treatment, and recurrence parameters in pre-treatment high-PNI recurrence groups (Figs. 4 and 5).

**Fig. 4:**
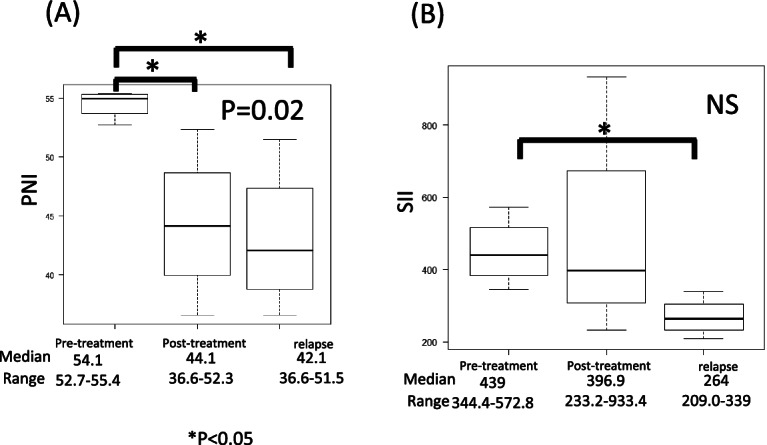
**A**, **B** Comparison of PNI and SII pre-treatment, post-treatment, and at relapse in the high-PNI group by Kruskal–Wallis test. PNI shows significant differences among groups. SII shows significant differences between pre-treatment and at relapse

**Fig. 5:**
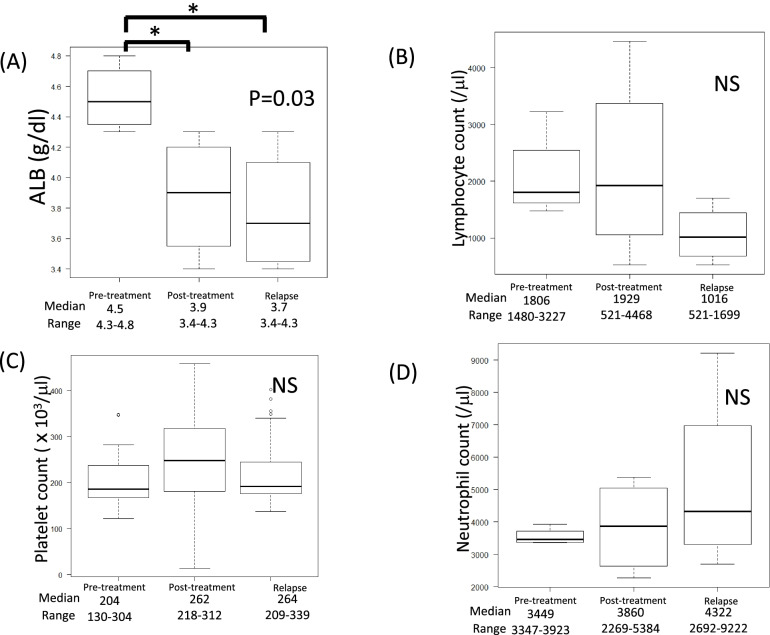
**A**–**D** Comparison of albumin concentration, lymphocyte count, platelet count and neutrophil count at pre-treatment, post-treatment, and relapse in the high-PNI group by Kruskal–Wallis test. Albumin concentration differs significantly among groups

For the high-PNI recurrence group, PNI and SII were compared before treatment, after treatment, and at recurrence. (*n* = 5). PNI showed significant differences between pre-treatment and post-treatment groups, and between pre-treatment and relapse groups (*p* = 0.02). SII differed significantly between pre-treatment and relapse groups, no other differences were evident between pre-treatment and relapse groups.

The same analysis of albumin and lymphocyte count, as components of PNI, showed no significant difference in lymphocyte count, but a significant difference in albumin (*p* = 0.03).

In addition to the lymphocyte count, the platelet and neutrophil counts, as components of SII, were also examined. No significant differences were apparent between pre- and post-treatment and at relapse.

### Comparison of pre-treatment, post-treatment, and recurrence parameters in the pre-treatment low-PNI recurrence groups (Figs. 6 and 7).

**Fig. 6:**
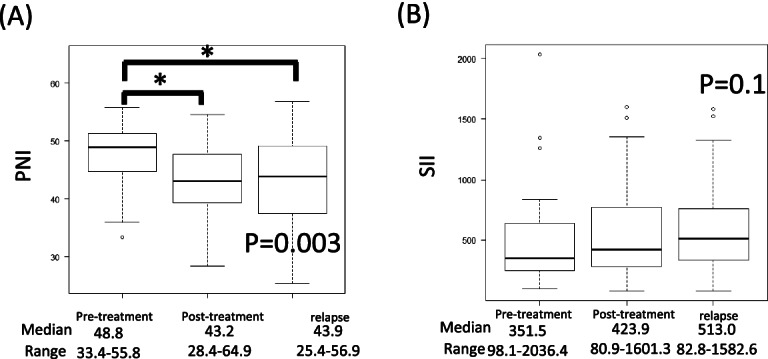
**A**, **B** Comparison of PNI and SII at pre-treatment, post-treatment, and relapse in the low-PNI group by Kruskal–Wallis test. PNI show significant differences among groups. SII show no significant differences between pre-treatment and relapse groups

**Fig. 7:**
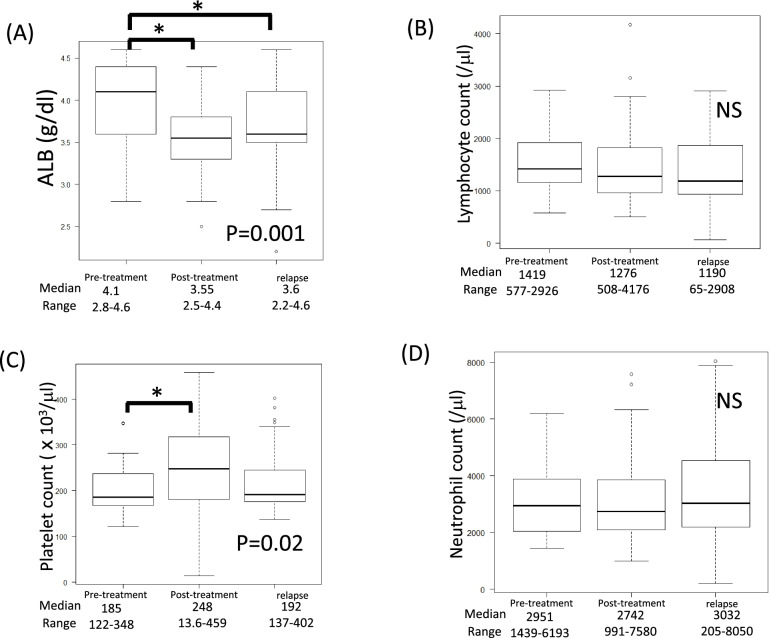
**A**–**D** Comparison of albumin concentration, lymphocyte count, platelet count and neutrophil count at pre-treatment, post-treatment, and relapse in the high-PNI group by Kruskal–Wallis test. Albumin concentration differs significantly among groups. Platelet count differs significantly between pre-treatment and post-treatment groups

For the low-PNI recurrence group, we compared PNI and SII among the pre-treatment, post-treatment, and relapse groups (*n* = 44). PNI showed significant differences between pre- and post-treatment groups, and between pre-treatment and relapse groups (*p* = 0.003). SII did not differ significantly among the pre-treatment, post-treatment, and relapse groups, but tended to increase.

The same analysis of albumin and lymphocyte count, as components of the PNI, showed no significant difference in lymphocyte count, but a significant difference in albumin (*p* = 0.001).

In addition to the lymphocyte count, both platelet and neutrophil counts, all of which are components of the SII, were also examined. Platelet count differed significantly between pre- and post-treatment (*p* = 0.02), but lymphocyte and neutrophil counts did not differ significantly between pre-treatment, post-treatment and relapse.

## Discussion

The present study retrospectively examined the utility of IBPS and SII as prognostic factors in oral cancer patients treated by surgery, combination surgery and RT, or SSIACRT. We revealed pre-treatment PNI to be a useful independent factor for predicting OS. To predict DFS, pre-treatment PNI and SII were useful independent factors.

PNI indicates not only the nutritional status, but also the immunological status of cancer patients. A low PNI implies a decrease in albumin and/or lymphocytes. Serum albumin concentration reflects the nutritional status and immune response of the organism. In addition, albumin organizes cellular growth and DNA stabilization, working like a buffer in biochemical reactions. Moreover, albumin maintains the regularity of sex hormones against cancers. Low serum albumin correlates with poor prognosis and worsened survival among patients with cancers [16]. Low lymphocyte levels thus suggest poor immune response, which can be related to worse prognosis [17]. Taken together, these lines of evidence indicate that malnutrition and lymphocytopenia may serve as indicators of a chronically impaired immune system.

Pre-treatment PNI has been shown to predict OS, and Onodera et al. proposed that a simplified PNI based only on serum albumin level and lymphocyte count can predict postoperative complications [12].

SII is a relatively new index that reflects inflammation status and is correlated with circulating tumor cells. A high SII has been associated with advanced clinicopathological characteristics and has been identified as a reliable prognostic factor for long-term survival in various malignant tumors [18]. SII has also been proposed to better reflect inflammatory status and prognosis than other inflammatory markers in many cancers [19–21]. A study by Jomrich et al. identified SII as a better prognostic factor than NLR or PLR in a receiver operating characteristic analysis of pancreatic ductal adenocarcinoma patients who underwent resection [22].

The components of SII are the neutrophil, lymphocyte, and platelet counts.

Neutrophils are induced by vascular endothelial growth factor and interleukin 8 expressed by cancer cells, which induce platelet-derived growth factor, fibroblast growth factor matrix metalloproteinase, and interleukin 6. These factors are associated with carcinogenesis, growth, and invasion in the tumor microenvironment [23, 24]. Neutrophils suppress many immune cells, like lymphocytes [25, 26]. An et al. explained that NLR reflected the balance between tumor growth environment and tumor immunity against tumors [27].

Lymphocytes are very important cellular components of the immune system that are crucial for the activation of effective antitumor responses [28], destruction of residual tumor cells, and the possibility of micrometastasis [29, 30]. Due to sustained activation of T cells in cancer patients, tumor-infiltrating T lymphocytes could help drive tumor cells towards apoptosis and, by presenting tumor-associated antigens to lymphocytes, lead to the death of cancer cells in response to chemoradiotherapy. Lymphocytes are therefore very important for improving adjuvant therapies and preventing tumor recurrence [31, 32].

Platelets can interact with and provide tumor cells with mechanical support [33]. Sabrkhany et al. confirmed that platelets can also promote cancer cell proliferation and metastasis by increasing angiogenesis and vessel permeability [34]. A study by Nieswandt et al. revealed that platelets also defend cancer cells from the host immune system by diminishing the cytotoxic activity of natural killer cells [35].

Taken together, a higher SII indicates an imbalance in the inflammatory response, which may lead to tumor invasion and poor prognosis, as reported by Lu et al. [8]. As a reflection of the cancer microenvironment, SII was considered to affect DFS more than OS in our study.

In this investigation, pre-treatment NLR, PLR, and LMR did not affect OS or DFS, but other reports have described these ratios as useful biomarkers in oral cancer [36–38]. We believe that further analysis is necessary by accumulating cases.

In both the high- and low-PNI groups of relapsed patients, albumin was found to be predominantly lower at post-treatment and at relapse compared to pre-treatment. Lymphocyte counts tended to decrease, but did not differ significantly, suggesting that albumin plays an important role in prognosis after treatment. Perioperative nutritional management may thus be important. Surgeons treating oral cancer would be well-advised to pay attention to patient nutritional conditions.

Malnutrition is thought to be present in 30–50% of head and neck cancer patients at the time of diagnosis [39]. Low levels of albumin post-treatment were considered to have important impacts on oral cancer patients from a prognostic perspective, as indicated by our data.

The downstream effects of treatment-related complications such as pain, fatigue, fibrosis, oral mucositis, and ongoing inflammation may worsen the malnutrition status following completion of therapy. For head and neck cancer patients, body weight loss continues and reaches a nadir approximately 6 months after therapy completion [40, 41]. Chang et al. reported that early recurrence can be predicted by malnourished status 3 months after treatment completion in patients with locally advanced oral cavity cancer treated by curative surgery and adjuvant chemoradiation [42]. That report mentioned that malnutrition exerts a significant negative effect on OS [42]. Our data suggested that continued malnutrition might offer a sign of relapse. Poor post-treatment patient conditions caused malnutrition, but collaboration with nutritionists and speech therapists before treatment to maintain nutrition and swallowing function may help to prevent that eventuality. We have previously reported that professional oral healthcare during SSIACRT had been promoted in our department, reducing opioid use and shortening the hospital stay [43]. Professions involved in the treatment of oral cancers should gather to discuss patient nutrition and physical condition during and after treatment. Bao et al. indicated that body mass index, albumin, PNI and nutritional risk index were of prognostic value in oral cancer patients and that more attention should be paid to nutrition support as a means of improving outcomes for oral cancer patients [44]. Given our results, improvement of OS in oral cancer patients will require improvements in PNI during the perioperative period to maximize post-treatment PNI and achieve good OS.

Our research indicated that PNI and SII are useful for predicting the prognosis of oral cancer patients. To predict long-term prognosis (i.e., OS), pre-treatment PNI is a positive factor related to better immunity and nutrition. In addition, for short-term prognosis (i.e., DFS), SII is a good factor reflecting the tumor and tumor microenvironment.

In this study, we analyzed all T factors without separating tumor sites, as the same method of analysis was applied in previous studies [13, 14, 25, 36]. Tumor size could conceivably be associated with worse nutritional status regardless of the location of oral cancer. In our study, the large number of T3 and T4 cases in the low-PNI group suggests that tumor size and nutritional immunity of oral cancer are related.

The main limitation of this study was that data from a single center were retrospectively analyzed. This study included a very heterogeneous sample of insufficient size (183 patients). Clinical differences were seen in age at first presentation, tumor location, tumor size, and presence of lymph node metastases, as well as the mix of treatment modalities: surgery (with or without RT or chemoradiotherapy) and SSIACRT. All these heterogeneities could have impacted the results for OS. Since the purpose of this study was to predict prognosis based on nutritional immunity and tumor immunity in PNI and SII, we believe that this study is significant, although future work should aim to exclude such sources of bias. Further study is needed to establish methods of IBPS for oral cancer treatment using evidence-based medicine.

## Conclusion

In this study, we investigated the usefulness of IBPS and SII as prognostic factors for OS and DFS in patients with oral cancer. The higher the pre-treatment PNI, the better the prognosis in terms of OS. Low pre-treatment PNI and high SII were associated with poor prognosis in terms of DFS. Our results suggest that PNI and SII may be useful in determining the optimal treatment for a given condition. In addition, incorporating PNI into clinical practice and improving nutritional status before and after treatment by healthcare providers may improve prognosis. We believe that PNI and SII represent potentially useful biomarkers for predicting prognosis in clinical settings.

## Data Availability

The datasets obtained and/or analyzed during the current study are available from the corresponding author on reasonable request.

## Declarations

### Competing of interests

None declared.
